# 3β-Chloro-6-[2-(2-cyano­acet­yl)hydrazin-1-yl­idene]-5α-cholestane

**DOI:** 10.1107/S1600536812009336

**Published:** 2012-03-14

**Authors:** Samina Khan Yusufzai, Hasnah Osman, Aisyah Saad Abdul Rahim, Suhana Arshad, Ibrahim Abdul Razak

**Affiliations:** a School of Chemical Sciences, Universiti Sains Malaysia, 11800 USM, Penang, Malaysia; b School of Pharmaceutical Sciences, Universiti Sains Malaysia, 11800 USM, Penang, Malaysia; c School of Physics, Universiti Sains Malaysia, 11800 USM, Penang, Malaysia

## Abstract

The asymmetric unit of the title compound, C_30_H_48_ClN_3_O, contains two mol­ecules, *A* and *B*. In both mol­ecules, the three cyclo­hexane rings in the steroid fused ring systems adopt chair conformations, while the cyclo­pentane rings adopt envelope and twist conformations in mol­ecules *A* and *B*, respectively. In mol­ecule *B*, the cyano group is disordered over two orientations with refined site-occupancies of 0.593 (8) and 0.407 (8). An intra­molecular C—H⋯N inter­action forms an *S*(10) ring in both mol­ecules. In the crystal, mol­ecules are linked by N—H⋯O, C—H⋯O and C—H⋯N inter­actions, resulting is chains propagating along the *a*-axis direction.

## Related literature

For related structures, see: Yusufzai *et al.* (2012 ▶); Ketuly *et al.* (2011 ▶). For ring conformations, see: Cremer & Pople (1975 ▶). For hydrogen-bond motifs, see: Bernstein *et al.* (1995 ▶). For the stability of the temperature controller used in the data collection, see: Cosier & Glazer (1986 ▶).
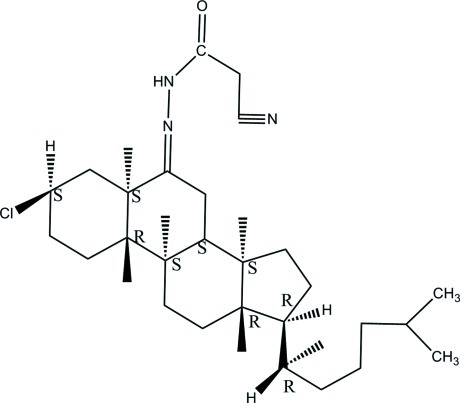


## Experimental

### 

#### Crystal data

C_30_H_48_ClN_3_O
*M* *_r_* = 502.16
Orthorhombic, 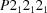

*a* = 11.1623 (2) Å
*b* = 19.7586 (3) Å
*c* = 26.4077 (4) Å
*V* = 5824.26 (16) Å^3^
*Z* = 8
Mo *K*α radiation
μ = 0.16 mm^−1^
*T* = 100 K
0.32 × 0.32 × 0.16 mm

#### Data collection

Bruker SMART APEXII CCD diffractometer
Absorption correction: multi-scan (*SADABS*; Bruker, 2009 ▶) *T*_min_ = 0.951, *T*_max_ = 0.976
51504 measured reflections
13243 independent reflections
12103 reflections with *I* > 2σ(*I*)
*R*_int_ = 0.042

#### Refinement

*R*[*F* ^2^ > 2σ(*F* ^2^)] = 0.104
*wR*(*F* ^2^) = 0.221
*S* = 1.18
13243 reflections
655 parameters
H-atom parameters constrained
Δρ_max_ = 0.50 e Å^−3^
Δρ_min_ = −0.52 e Å^−3^
Absolute structure: Flack (1983 ▶), 5882 Friedel pairs
Flack parameter: 0.05 (11)

### 

Data collection: *APEX2* (Bruker, 2009 ▶); cell refinement: *SAINT* (Bruker, 2009 ▶); data reduction: *SAINT*; program(s) used to solve structure: *SHELXTL* (Sheldrick, 2008 ▶); program(s) used to refine structure: *SHELXTL*; molecular graphics: *SHELXTL*; software used to prepare material for publication: *SHELXTL* and *PLATON* (Spek, 2009 ▶).

## Supplementary Material

Crystal structure: contains datablock(s) global, I. DOI: 10.1107/S1600536812009336/hb6661sup1.cif

Structure factors: contains datablock(s) I. DOI: 10.1107/S1600536812009336/hb6661Isup2.hkl

Additional supplementary materials:  crystallographic information; 3D view; checkCIF report

## Figures and Tables

**Table 1:** Hydrogen-bond geometry (Å, °)

*D*—H⋯*A*	*D*—H	H⋯*A*	*D*⋯*A*	*D*—H⋯*A*
C4*B*—H4*BA*⋯N3*B*	0.99	2.58	3.469 (10)	149
N2*A*—H1*NA*⋯O1*B*^i^	0.85	2.03	2.870 (6)	168
N2*B*—H1*NB*⋯O1*A*^ii^	0.85	2.05	2.894 (6)	171
C1*A*—H1*AB*⋯O1*B*^i^	0.99	2.41	3.362 (7)	162
C1*B*—H1*BB*⋯O1*A*^ii^	0.99	2.44	3.352 (6)	153
C4*A*—H4*AB*⋯N3*B*^iii^	0.99	2.49	3.476 (9)	173
C19*A*—H19*A*⋯N3*B*^iii^	0.99	2.57	3.513 (11)	160

Symmetry codes: (i) 
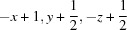
; (ii) 
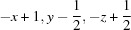
; (iii) 
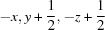
.

**Fig. 1.**
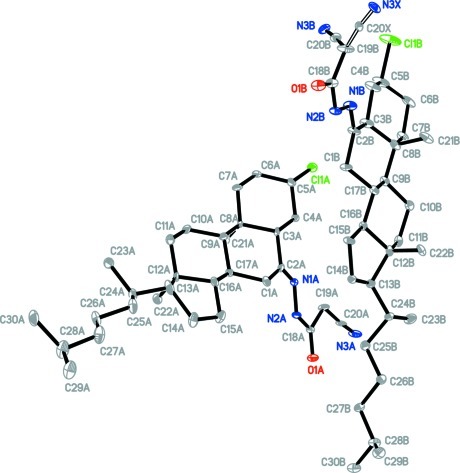
The molecular structure of the title compound, showing 30% probability displacement ellipsoids. H-atoms are omitted for clarity. Disordered components are shown.

**Fig. 2.**
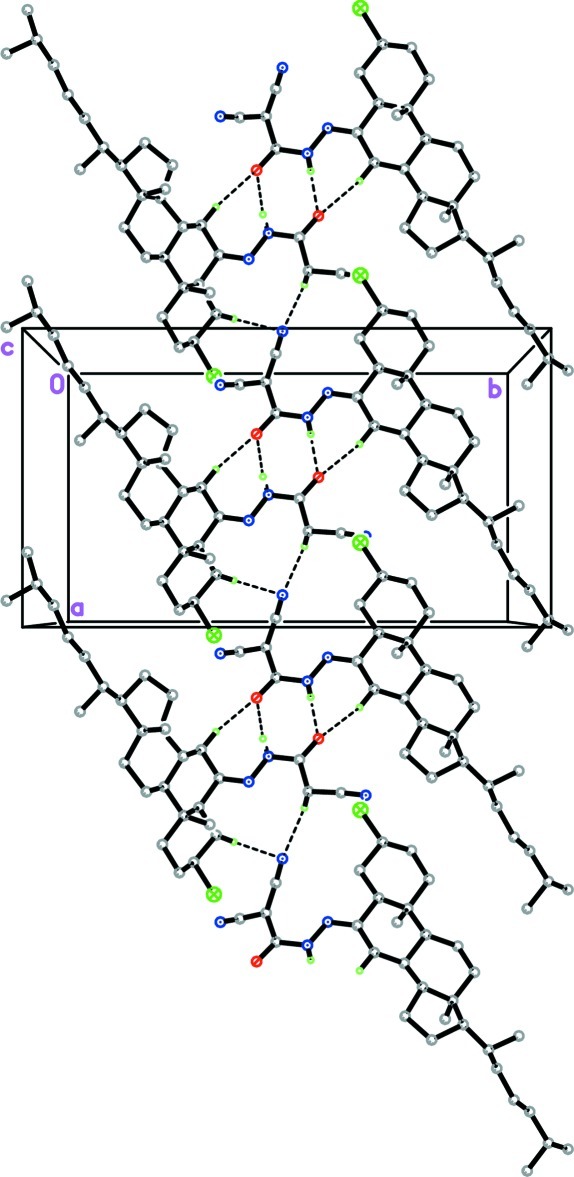
The crystal packing of the title compound. Only major disordered components is shown.

## supplementary crystallographic information

### Comment

The study being reported in this paper is a part of our on going effort towards the synthesis of modified steroids, which may be biologically active. In continuation of our previous work (Yusufzai *et al.*, 2012) we report the synthesis of 3β-chloro-6-[2-(2-cyanoacetyl)hydrazin-1-ylidene]-5α-cholestane which corresponds to the molecular formula, C_30_H_48_N_3_OCl.

The asymmetric unit of the title compound (Fig. 1), consists of two crystallographically independent molecules *A* and *B*. The bond lengths are comparable to those in related structures (Yusufzai *et al.*, 2012; Ketuly *et al.*, 2011). In molecule *B*, the nitrile group is disordered over two positions with refined site-occupancies of 0.593 (8): 0.407 (8) ratio. The cyclopentane ring of the steroid fused ring system in both molecules adopts a different ring conformation (Cremer & Pople, 1975). In molecule *A*, it is in twist conformation where the cyclopentane (C12A–C16A) ring is twisted about C16A–C12A bonds, with puckering parameters Q= 0.472 (6) Å and φ= 348.8 (8)°. Meanwhile, the cyclopentane (C12B–C16B) ring of molecule *B* is in envelope conformation with puckering parameters Q= 0.456 (5) Å and φ= 353.6 (7)° with atom C12B at the flap. In addition, the three cyclohexane rings in the steroid fused ring system for both molecules adopt a chair conformation [Molecule *A* (C1A–C3A/C8A/C9A/C17A):(C3A–C8A):(C9A–C12A/C16A/C17A); Q= 0.583 (6):0.562 (3):0.588 (6) Å, Θ= 172.4 (6):178.7 (3):175.7 (6)° and Φ= 305 (4):232 (10):120 (8)°; Molecule *B* (C1B–C3B/C8B/C9B/C17B): (C3B–C8B):(C9B–C12B/C16B/C17B); Q= 0.549 (6):0.595 (6):0.568 (5) Å, Θ= 172.2 (6):178.9 (6):174.7 (5)° and Φ= 30 (4):258 (23):31 (6)°]. Furthermore, an intramolecular C4B—H4BA···N3B hydrogen bond is observed in *B* and forms an *S*(10) ring motif (Bernstein *et al.*, 1995).

There are nine chiral centres presented in molecule *A* and *B*. In each molecules, the centers exhibit the following relative chiralities: C3A/C3B = *S*; C5A/C5B = *S*; C8A/C8B = *R*; C9A/C9B = *S*; C12A/C12B = *R*; C13A/C13B = *R*; C16A/C16B = *S*; C17A/C17B = *S* and C24A/C24B = *R*.

The crystal packing is shown in Fig. 2. N—H···O (Table 1) hydrogen bonds generate *R*^2^_2_(8) ring motifs, sandwiched by two *R*^1^_2_(7) ring motifs when combined with C—H···O (Table 1) hydrogen bonds. In addition, C—H···N (Table 1) interactions form *R*^1^_2_(10) ring motifs. These ring motifs link the molecules into chains along *a*-axis.

### Experimental

To a solution of steroidal ketone 3β-chloro-5α-cholestan-6-one (5 mmol), in absolute ethanol (10 ml) was added cyanoacetohydrazide (10 mmol) followed by few drops of triethylamine. The reaction mixture was refluxed for 24 hrs. The progress of reaction was monitored by thin layer chromatography. After completion of reaction, reaction mixture was concentrated under reduce pressure. The obtained solid, was extracted with ether and ethereal layer was washed with water, NaHCO_3_ solution (5%), again with water and dried over anhydrous sodium sulfate. The solvent was evaporated and the product was recrystallized from ethanol to give compound as colourless blocks.

### Refinement

The nitrile group of molecule *B* was disordered over two positions with refined site-occupancies of 0.593 (8): 0.407 (8) ratio. N-bound H atoms was located from the difference fourier map and was fixed at its found location using riding model with *U*_iso_(H) = 1.5 *U*_eq_(N) [N–H = 0.8537 and 0.8549 Å]. The remaining H atoms were positioned geometrically [C–H = 0.98–1.0 Å] and refined using a riding model with *U*_iso_(H) = 1.2 or 1.5 *U*_eq_(C). A rotating group model was applied to the methyl groups. The same *U^ij^* parameters were used for atoms pair C28A and C29A. 5882 Friedel pairs were used to determine the absolute configuration.

### Crystal data

**Table:**

C_30_H_48_ClN_3_O	*F*(000) = 2192
*M**_r_* = 502.16	*D*_x_ = 1.145 Mg m^−^^3^
Orthorhombic, *P*2_1_2_1_2_1_	Mo *K*α radiation, λ = 0.71073 Å
Hall symbol: P 2ac 2ab	Cell parameters from 9967 reflections
*a* = 11.1623 (2) Å	θ = 2.2–28.1°
*b* = 19.7586 (3) Å	µ = 0.16 mm^−^^1^
*c* = 26.4077 (4) Å	*T* = 100 K
*V* = 5824.26 (16) Å^3^	Block, colourless
*Z* = 8	0.32 × 0.32 × 0.16 mm

### Data collection

**Table:**

Bruker SMART APEXII CCD diffractometer	13243 independent reflections
Radiation source: fine-focus sealed tube	12103 reflections with *I* > 2σ(*I*)
Graphite monochromator	*R*_int_ = 0.042
φ and ω scans	θ_max_ = 27.5°, θ_min_ = 1.3°
Absorption correction: multi-scan (*SADABS*; Bruker, 2009)	*h* = −14→14
*T*_min_ = 0.951, *T*_max_ = 0.976	*k* = −25→25
51504 measured reflections	*l* = −34→34

### Refinement

**Table:**

Refinement on *F*^2^	Hydrogen site location: inferred from neighbouring sites
Least-squares matrix: full	H-atom parameters constrained
*R*[*F*^2^ > 2σ(*F*^2^)] = 0.104	*w* = 1/[σ^2^(*F*_o_^2^) + (0.*P*)^2^ + 25.2717*P*] where *P* = (*F*_o_^2^ + 2*F*_c_^2^)/3
*wR*(*F*^2^) = 0.221	(Δ/σ)_max_ < 0.001
*S* = 1.18	Δρ_max_ = 0.50 e Å^−^^3^
13243 reflections	Δρ_min_ = −0.52 e Å^−^^3^
655 parameters	Extinction correction: *SHELXL*, Fc^*^=kFc[1+0.001xFc^2^λ^3^/sin(2θ)]^-1/4^
0 restraints	Extinction coefficient: 0.00130 (14)
Primary atom site location: structure-invariant direct methods	Absolute structure: Flack (1983), 5882 Friedel pairs
Secondary atom site location: difference Fourier map	Flack parameter: 0.05 (11)

### Special details

**Table:**

Experimental. The crystal was placed in the cold stream of an Oxford Cryosystems Cobra open-flow nitrogen cryostat (Cosier & Glazer, 1986) operating at 100.0 (1) K.
Geometry. All e.s.d.'s (except the e.s.d. in the dihedral angle between two l.s. planes) are estimated using the full covariance matrix. The cell e.s.d.'s are taken into account individually in the estimation of e.s.d.'s in distances, angles and torsion angles; correlations between e.s.d.'s in cell parameters are only used when they are defined by crystal symmetry. An approximate (isotropic) treatment of cell e.s.d.'s is used for estimating e.s.d.'s involving l.s. planes.
Refinement. Refinement of *F*^2^ against ALL reflections. The weighted *R*-factor *wR* and goodness of fit *S* are based on *F*^2^, conventional *R*-factors *R* are based on *F*, with *F* set to zero for negative *F*^2^. The threshold expression of *F*^2^ > σ(*F*^2^) is used only for calculating *R*-factors(gt) *etc*. and is not relevant to the choice of reflections for refinement. *R*-factors based on *F*^2^ are statistically about twice as large as those based on *F*, and *R*- factors based on ALL data will be even larger.

### Fractional atomic coordinates and isotropic or equivalent isotropic displacement parameters (Å^2^)

**Table:**

	*x*	*y*	*z*	*U*_iso_*/*U*_eq_	Occ. (<1)
Cl1A	−0.04761 (13)	0.83894 (7)	0.25082 (6)	0.0339 (3)	
O1A	0.5580 (3)	1.06640 (17)	0.20162 (15)	0.0272 (8)	
N1A	0.3929 (4)	0.9157 (2)	0.20369 (15)	0.0195 (8)	
N2A	0.4907 (4)	0.9587 (2)	0.19816 (16)	0.0218 (9)	
H1NA	0.5596	0.9465	0.1874	0.033*	
N3A	0.3345 (5)	1.1702 (3)	0.2533 (2)	0.0450 (15)	
C1A	0.5176 (5)	0.8138 (3)	0.18260 (18)	0.0233 (11)	
H1AA	0.5357	0.7801	0.2092	0.028*	
H1AB	0.5863	0.8453	0.1801	0.028*	
C2A	0.4073 (5)	0.8523 (2)	0.19657 (17)	0.0187 (10)	
C3A	0.2960 (5)	0.8082 (2)	0.19943 (18)	0.0216 (10)	
H3AA	0.3147	0.7695	0.2225	0.026*	
C4A	0.1858 (5)	0.8447 (2)	0.22126 (18)	0.0218 (10)	
H4AA	0.2051	0.8629	0.2552	0.026*	
H4AB	0.1638	0.8832	0.1991	0.026*	
C5A	0.0813 (5)	0.7960 (3)	0.2252 (2)	0.0266 (12)	
H5AA	0.1039	0.7580	0.2483	0.032*	
C6A	0.0511 (6)	0.7672 (3)	0.1733 (2)	0.0306 (13)	
H6AA	0.0252	0.8041	0.1505	0.037*	
H6AB	−0.0157	0.7345	0.1764	0.037*	
C7A	0.1618 (5)	0.7316 (3)	0.1510 (2)	0.0316 (13)	
H7AA	0.1831	0.6928	0.1729	0.038*	
H7AB	0.1408	0.7134	0.1172	0.038*	
C8A	0.2721 (5)	0.7778 (3)	0.14556 (18)	0.0250 (12)	
C9A	0.3842 (5)	0.7368 (3)	0.1301 (2)	0.0264 (12)	
H9AA	0.3934	0.7001	0.1559	0.032*	
C10A	0.3716 (6)	0.7015 (3)	0.0785 (2)	0.0405 (16)	
H10A	0.3637	0.7361	0.0516	0.049*	
H10B	0.2980	0.6736	0.0784	0.049*	
C11A	0.4810 (6)	0.6560 (3)	0.0670 (2)	0.0434 (17)	
H11A	0.4825	0.6182	0.0916	0.052*	
H11B	0.4714	0.6363	0.0328	0.052*	
C12A	0.6011 (5)	0.6934 (2)	0.06944 (17)	0.0221 (11)	
C13A	0.7141 (7)	0.6488 (3)	0.0738 (2)	0.0394 (16)	
H13A	0.6950	0.6122	0.0987	0.047*	
C14A	0.8061 (6)	0.6973 (3)	0.1001 (2)	0.0358 (14)	
H14A	0.8537	0.6723	0.1257	0.043*	
H14B	0.8616	0.7169	0.0748	0.043*	
C15A	0.7304 (6)	0.7543 (3)	0.1260 (2)	0.0340 (13)	
H15A	0.7534	0.7599	0.1620	0.041*	
H15B	0.7407	0.7981	0.1083	0.041*	
C16A	0.6038 (6)	0.7290 (2)	0.12135 (19)	0.0275 (12)	
H16A	0.5958	0.6922	0.1471	0.033*	
C17A	0.4992 (5)	0.7774 (2)	0.13125 (18)	0.0214 (10)	
H17A	0.4967	0.8120	0.1037	0.026*	
C18A	0.4770 (5)	1.0254 (2)	0.20651 (19)	0.0209 (10)	
C19A	0.3534 (5)	1.0449 (3)	0.2252 (2)	0.0300 (13)	
H19A	0.2942	1.0361	0.1981	0.036*	
H19B	0.3321	1.0163	0.2546	0.036*	
C20A	0.3463 (5)	1.1162 (3)	0.2400 (2)	0.0316 (13)	
C21A	0.2448 (5)	0.8344 (3)	0.10696 (18)	0.0277 (12)	
H21A	0.2148	0.8143	0.0755	0.042*	
H21B	0.3183	0.8599	0.0999	0.042*	
H21C	0.1842	0.8650	0.1210	0.042*	
C22A	0.6114 (5)	0.7438 (3)	0.02495 (19)	0.0275 (12)	
H22A	0.5497	0.7788	0.0283	0.041*	
H22B	0.6001	0.7196	−0.0071	0.041*	
H22C	0.6908	0.7649	0.0254	0.041*	
C23A	0.6780 (7)	0.5678 (3)	0.0007 (2)	0.0429 (17)	
H23A	0.7197	0.5419	−0.0256	0.064*	
H23B	0.6146	0.5951	−0.0148	0.064*	
H23C	0.6425	0.5365	0.0253	0.064*	
C24A	0.7676 (6)	0.6144 (3)	0.02777 (19)	0.0294 (12)	
H24A	0.7929	0.6504	0.0034	0.035*	
C25A	0.8795 (7)	0.5741 (3)	0.0433 (2)	0.0374 (15)	
H25A	0.8542	0.5374	0.0664	0.045*	
H25B	0.9329	0.6046	0.0627	0.045*	
C26A	0.9527 (6)	0.5425 (3)	0.0000 (2)	0.0325 (13)	
H26A	0.9039	0.5072	−0.0168	0.039*	
H26B	0.9714	0.5778	−0.0254	0.039*	
C27A	1.0694 (7)	0.5109 (3)	0.0189 (2)	0.0410 (16)	
H27A	1.1161	0.5463	0.0367	0.049*	
H27B	1.0495	0.4755	0.0441	0.049*	
C28A	1.1483 (6)	0.4797 (3)	−0.0216 (3)	0.0432 (12)	
H28A	1.1648	0.5150	−0.0478	0.052*	
C29A	1.2657 (6)	0.4581 (3)	0.0009 (3)	0.0432 (12)	
H29A	1.3055	0.4974	0.0162	0.065*	
H29B	1.3169	0.4392	−0.0257	0.065*	
H29C	1.2515	0.4238	0.0270	0.065*	
C30A	1.0869 (7)	0.4199 (3)	−0.0476 (2)	0.0413 (16)	
H30A	1.1392	0.4021	−0.0742	0.062*	
H30B	1.0111	0.4349	−0.0625	0.062*	
H30C	1.0712	0.3843	−0.0226	0.062*	
Cl1B	−0.30342 (14)	0.65408 (10)	0.42098 (10)	0.0666 (7)	
O1B	0.2818 (4)	0.43353 (19)	0.34895 (17)	0.0319 (9)	
N1B	0.1279 (4)	0.5859 (2)	0.36554 (19)	0.0276 (10)	
N2B	0.2194 (4)	0.5423 (2)	0.35160 (18)	0.0240 (9)	
H1NB	0.2805	0.5496	0.3329	0.036*	
N3B	−0.1078 (8)	0.4889 (4)	0.3466 (3)	0.036 (2)	0.593 (8)
C20B	−0.0208 (9)	0.4748 (4)	0.3670 (4)	0.027 (2)	0.593 (8)
N3X	0.1102 (11)	0.3588 (6)	0.4478 (4)	0.033 (3)	0.407 (8)
C20X	0.1042 (12)	0.3992 (6)	0.4172 (5)	0.024 (3)	0.407 (8)
C1B	0.2503 (4)	0.6858 (2)	0.3379 (2)	0.0232 (11)	
H1BA	0.2336	0.6997	0.3026	0.028*	
H1BB	0.3194	0.6544	0.3374	0.028*	
C2B	0.1429 (4)	0.6494 (3)	0.3588 (2)	0.0246 (11)	
C3B	0.0363 (5)	0.6942 (3)	0.3708 (2)	0.0317 (13)	
H3BA	0.0108	0.7142	0.3378	0.038*	
C4B	−0.0719 (5)	0.6534 (3)	0.3899 (3)	0.0378 (15)	
H4BA	−0.0910	0.6166	0.3658	0.045*	
H4BB	−0.0534	0.6328	0.4232	0.045*	
C5B	−0.1784 (6)	0.7016 (3)	0.3948 (3)	0.0460 (19)	
H5BA	−0.2009	0.7184	0.3604	0.055*	
C6B	−0.1530 (5)	0.7614 (3)	0.4286 (2)	0.0364 (15)	
H6BA	−0.1402	0.7456	0.4637	0.044*	
H6BB	−0.2228	0.7923	0.4285	0.044*	
C7B	−0.0414 (5)	0.7996 (3)	0.4102 (2)	0.0300 (12)	
H7BA	−0.0593	0.8207	0.3771	0.036*	
H7BB	−0.0233	0.8364	0.4345	0.036*	
C8B	0.0696 (5)	0.7547 (3)	0.4047 (2)	0.0275 (11)	
C9B	0.1736 (5)	0.7944 (2)	0.3787 (2)	0.0222 (11)	
H9BA	0.1433	0.8078	0.3445	0.027*	
C10B	0.2100 (5)	0.8608 (2)	0.4055 (2)	0.0232 (10)	
H10C	0.1398	0.8914	0.4067	0.028*	
H10D	0.2331	0.8503	0.4409	0.028*	
C11B	0.3148 (5)	0.8978 (2)	0.3793 (2)	0.0249 (11)	
H11C	0.2878	0.9145	0.3458	0.030*	
H11D	0.3381	0.9376	0.3999	0.030*	
C12B	0.4238 (5)	0.8522 (2)	0.37214 (18)	0.0215 (10)	
C13B	0.5244 (5)	0.8768 (2)	0.33587 (19)	0.0228 (11)	
H13B	0.4844	0.8958	0.3051	0.027*	
C14B	0.5878 (5)	0.8101 (3)	0.3195 (2)	0.0248 (11)	
H14C	0.6123	0.8126	0.2835	0.030*	
H14D	0.6599	0.8021	0.3405	0.030*	
C15B	0.4952 (5)	0.7525 (3)	0.3274 (2)	0.0264 (11)	
H15C	0.4831	0.7266	0.2957	0.032*	
H15D	0.5217	0.7211	0.3543	0.032*	
C16B	0.3805 (5)	0.7892 (2)	0.34283 (18)	0.0181 (10)	
H16B	0.3440	0.8065	0.3108	0.022*	
C17B	0.2834 (5)	0.7489 (2)	0.36931 (19)	0.0203 (10)	
H17B	0.3150	0.7336	0.4028	0.024*	
C18B	0.2049 (5)	0.4753 (3)	0.3603 (2)	0.0253 (11)	
C19B	0.0894 (5)	0.4542 (3)	0.3861 (3)	0.0385 (15)	
H19C	0.0886	0.4041	0.3872	0.046*	0.593 (8)
H19D	0.0939	0.4701	0.4216	0.046*	0.593 (8)
H19E	0.0586	0.4926	0.4063	0.046*	0.407 (8)
H19F	0.0289	0.4432	0.3599	0.046*	0.407 (8)
C21B	0.1082 (6)	0.7304 (3)	0.4578 (2)	0.0390 (15)	
H21D	0.0392	0.7104	0.4752	0.058*	
H21E	0.1382	0.7689	0.4774	0.058*	
H21F	0.1716	0.6964	0.4544	0.058*	
C22B	0.4781 (5)	0.8330 (3)	0.42409 (19)	0.0287 (12)	
H22D	0.5027	0.8742	0.4419	0.043*	
H22E	0.5480	0.8038	0.4190	0.043*	
H22F	0.4181	0.8089	0.4443	0.043*	
C23B	0.5555 (6)	0.9928 (3)	0.3767 (3)	0.0383 (14)	
H23D	0.6158	1.0283	0.3814	0.057*	
H23E	0.5196	0.9814	0.4095	0.057*	
H23F	0.4930	1.0090	0.3536	0.057*	
C24B	0.6150 (5)	0.9299 (3)	0.3543 (2)	0.0255 (11)	
H24B	0.6650	0.9086	0.3814	0.031*	
C25B	0.6988 (5)	0.9511 (3)	0.3109 (2)	0.0275 (12)	
H25C	0.7211	0.9102	0.2913	0.033*	
H25D	0.6544	0.9816	0.2878	0.033*	
C26B	0.8134 (5)	0.9867 (3)	0.3278 (2)	0.0272 (12)	
H26C	0.7940	1.0190	0.3553	0.033*	
H26D	0.8699	0.9528	0.3416	0.033*	
C27B	0.8733 (5)	1.0243 (2)	0.2851 (2)	0.0227 (11)	
H27C	0.8150	1.0568	0.2706	0.027*	
H27D	0.8942	0.9915	0.2582	0.027*	
C28B	0.9876 (5)	1.0635 (2)	0.3002 (2)	0.0272 (12)	
H28B	0.9679	1.0921	0.3303	0.033*	
C29B	1.0886 (5)	1.0163 (3)	0.3150 (3)	0.0368 (14)	
H29D	1.0652	0.9904	0.3450	0.055*	
H29E	1.1605	1.0429	0.3225	0.055*	
H29F	1.1053	0.9852	0.2870	0.055*	
C30B	1.0274 (5)	1.1102 (3)	0.2579 (2)	0.0329 (13)	
H30D	0.9590	1.1372	0.2464	0.049*	
H30E	1.0584	1.0832	0.2297	0.049*	
H30F	1.0905	1.1404	0.2704	0.049*	

### Atomic displacement parameters (Å^2^)

**Table:**

	*U*^11^	*U*^22^	*U*^33^	*U*^12^	*U*^13^	*U*^23^
Cl1A	0.0317 (7)	0.0336 (7)	0.0364 (7)	−0.0058 (6)	0.0101 (6)	−0.0085 (6)
O1A	0.0176 (18)	0.0201 (17)	0.044 (2)	−0.0037 (15)	0.0072 (17)	−0.0058 (16)
N1A	0.020 (2)	0.0201 (19)	0.0183 (19)	−0.0047 (17)	0.0031 (17)	−0.0016 (15)
N2A	0.018 (2)	0.0204 (19)	0.026 (2)	−0.0060 (17)	0.0053 (18)	−0.0007 (16)
N3A	0.034 (3)	0.031 (3)	0.070 (4)	−0.013 (2)	0.031 (3)	−0.017 (3)
C1A	0.030 (3)	0.023 (2)	0.017 (2)	0.003 (2)	0.003 (2)	0.0035 (18)
C2A	0.026 (3)	0.017 (2)	0.013 (2)	−0.002 (2)	−0.0014 (19)	−0.0022 (17)
C3A	0.031 (3)	0.019 (2)	0.015 (2)	−0.006 (2)	0.004 (2)	−0.0085 (18)
C4A	0.029 (3)	0.019 (2)	0.017 (2)	−0.006 (2)	0.005 (2)	−0.0017 (18)
C5A	0.032 (3)	0.022 (2)	0.025 (3)	−0.005 (2)	0.009 (2)	−0.001 (2)
C6A	0.031 (3)	0.031 (3)	0.030 (3)	−0.017 (3)	0.013 (3)	−0.007 (2)
C7A	0.029 (3)	0.026 (3)	0.040 (3)	−0.017 (2)	0.009 (3)	−0.009 (2)
C8A	0.030 (3)	0.029 (3)	0.016 (2)	−0.016 (2)	0.011 (2)	−0.014 (2)
C9A	0.024 (3)	0.022 (2)	0.032 (3)	−0.009 (2)	0.008 (2)	−0.009 (2)
C10A	0.032 (3)	0.049 (4)	0.041 (3)	−0.020 (3)	0.018 (3)	−0.029 (3)
C11A	0.050 (4)	0.038 (3)	0.042 (3)	−0.015 (3)	0.026 (3)	−0.028 (3)
C12A	0.033 (3)	0.022 (2)	0.011 (2)	−0.002 (2)	0.004 (2)	−0.0059 (18)
C13A	0.070 (5)	0.023 (3)	0.026 (3)	0.011 (3)	0.020 (3)	0.000 (2)
C14A	0.040 (4)	0.034 (3)	0.033 (3)	0.018 (3)	−0.003 (3)	−0.013 (2)
C15A	0.036 (3)	0.032 (3)	0.035 (3)	0.004 (3)	−0.006 (3)	−0.013 (2)
C16A	0.046 (4)	0.017 (2)	0.019 (2)	−0.008 (2)	0.011 (2)	−0.0007 (19)
C17A	0.027 (3)	0.020 (2)	0.018 (2)	−0.005 (2)	0.005 (2)	−0.0011 (18)
C18A	0.020 (3)	0.020 (2)	0.022 (2)	−0.004 (2)	0.002 (2)	0.0003 (18)
C19A	0.021 (3)	0.020 (2)	0.048 (3)	−0.009 (2)	0.009 (3)	−0.010 (2)
C20A	0.020 (3)	0.030 (3)	0.045 (3)	−0.012 (2)	0.020 (3)	−0.009 (2)
C21A	0.034 (3)	0.033 (3)	0.016 (2)	−0.011 (2)	0.006 (2)	−0.005 (2)
C22A	0.030 (3)	0.028 (3)	0.025 (3)	0.002 (2)	0.004 (2)	−0.002 (2)
C23A	0.059 (5)	0.029 (3)	0.041 (3)	−0.016 (3)	0.023 (3)	−0.017 (3)
C24A	0.044 (4)	0.027 (3)	0.018 (2)	−0.001 (3)	0.005 (2)	−0.001 (2)
C25A	0.060 (4)	0.033 (3)	0.019 (3)	0.010 (3)	0.003 (3)	−0.004 (2)
C26A	0.047 (4)	0.031 (3)	0.019 (2)	0.014 (3)	0.008 (3)	0.000 (2)
C27A	0.061 (5)	0.038 (3)	0.024 (3)	0.019 (3)	−0.010 (3)	−0.001 (2)
C28A	0.043 (3)	0.043 (2)	0.044 (3)	0.024 (2)	−0.010 (2)	−0.013 (2)
C29A	0.043 (3)	0.043 (2)	0.044 (3)	0.024 (2)	−0.010 (2)	−0.013 (2)
C30A	0.057 (5)	0.036 (3)	0.031 (3)	0.007 (3)	0.011 (3)	−0.011 (3)
Cl1B	0.0194 (7)	0.0534 (10)	0.1270 (19)	0.0128 (8)	0.0330 (10)	0.0390 (12)
O1B	0.021 (2)	0.0231 (18)	0.052 (3)	0.0050 (16)	0.0054 (19)	0.0013 (17)
N1B	0.012 (2)	0.024 (2)	0.047 (3)	0.0063 (18)	0.004 (2)	0.005 (2)
N2B	0.014 (2)	0.020 (2)	0.038 (2)	−0.0005 (17)	0.0044 (19)	0.0048 (18)
N3B	0.036 (5)	0.030 (4)	0.042 (5)	−0.012 (4)	−0.003 (4)	−0.009 (4)
C20B	0.023 (5)	0.023 (4)	0.035 (5)	−0.002 (4)	0.005 (4)	−0.004 (4)
N3X	0.031 (7)	0.044 (7)	0.022 (5)	−0.006 (6)	0.000 (5)	0.017 (5)
C20X	0.027 (7)	0.025 (6)	0.020 (6)	−0.003 (5)	0.014 (5)	−0.011 (5)
C1B	0.009 (2)	0.017 (2)	0.043 (3)	0.0037 (18)	0.010 (2)	0.000 (2)
C2B	0.007 (2)	0.024 (2)	0.043 (3)	0.006 (2)	0.010 (2)	0.008 (2)
C3B	0.021 (3)	0.021 (2)	0.053 (4)	0.008 (2)	0.012 (3)	0.011 (2)
C4B	0.015 (3)	0.037 (3)	0.062 (4)	0.015 (2)	0.016 (3)	0.023 (3)
C5B	0.025 (3)	0.036 (3)	0.078 (5)	0.014 (3)	0.027 (3)	0.024 (3)
C6B	0.022 (3)	0.043 (3)	0.044 (3)	0.016 (3)	0.020 (3)	0.018 (3)
C7B	0.024 (3)	0.031 (3)	0.035 (3)	0.015 (2)	0.011 (2)	0.008 (2)
C8B	0.021 (3)	0.035 (3)	0.027 (3)	0.007 (2)	0.008 (2)	0.008 (2)
C9B	0.025 (3)	0.018 (2)	0.024 (2)	0.008 (2)	0.005 (2)	0.0039 (19)
C10B	0.021 (3)	0.024 (2)	0.025 (2)	0.007 (2)	0.004 (2)	−0.002 (2)
C11B	0.034 (3)	0.019 (2)	0.022 (2)	0.009 (2)	−0.002 (2)	−0.0027 (19)
C12B	0.031 (3)	0.017 (2)	0.016 (2)	0.006 (2)	0.000 (2)	−0.0022 (18)
C13B	0.027 (3)	0.019 (2)	0.022 (2)	−0.005 (2)	−0.002 (2)	0.0018 (18)
C14B	0.023 (3)	0.027 (3)	0.025 (2)	−0.006 (2)	0.008 (2)	−0.007 (2)
C15B	0.021 (3)	0.020 (2)	0.038 (3)	−0.001 (2)	0.014 (2)	−0.002 (2)
C16B	0.023 (3)	0.014 (2)	0.018 (2)	0.0027 (19)	0.002 (2)	0.0004 (17)
C17B	0.019 (2)	0.018 (2)	0.024 (2)	0.007 (2)	0.005 (2)	0.0025 (19)
C18B	0.017 (2)	0.022 (2)	0.037 (3)	0.002 (2)	−0.003 (2)	0.005 (2)
C19B	0.019 (3)	0.026 (3)	0.070 (4)	−0.001 (2)	0.011 (3)	0.016 (3)
C21B	0.025 (3)	0.050 (4)	0.042 (3)	0.006 (3)	0.010 (3)	0.023 (3)
C22B	0.026 (3)	0.041 (3)	0.019 (2)	0.005 (2)	0.005 (2)	0.005 (2)
C23B	0.037 (3)	0.026 (3)	0.052 (4)	−0.006 (3)	0.002 (3)	−0.011 (3)
C24B	0.028 (3)	0.021 (2)	0.028 (3)	−0.010 (2)	0.003 (2)	−0.003 (2)
C25B	0.030 (3)	0.029 (3)	0.024 (3)	−0.004 (2)	−0.005 (2)	0.004 (2)
C26B	0.023 (3)	0.032 (3)	0.027 (3)	0.001 (2)	−0.004 (2)	0.000 (2)
C27B	0.019 (3)	0.018 (2)	0.030 (3)	−0.001 (2)	−0.007 (2)	0.0033 (19)
C28B	0.021 (3)	0.018 (2)	0.043 (3)	0.003 (2)	0.004 (2)	0.000 (2)
C29B	0.020 (3)	0.043 (3)	0.048 (4)	0.008 (3)	0.004 (3)	−0.011 (3)
C30B	0.023 (3)	0.022 (2)	0.054 (4)	0.001 (2)	0.006 (3)	0.000 (2)

### Geometric parameters (Å, º)

**Table:**

Cl1A—C5A	1.803 (6)	N1B—C2B	1.279 (7)
O1A—C18A	1.221 (6)	N1B—N2B	1.385 (6)
N1A—C2A	1.277 (6)	N2B—C18B	1.354 (6)
N1A—N2A	1.391 (6)	N2B—H1NB	0.8549
N2A—C18A	1.345 (6)	N3B—C20B	1.145 (13)
N2A—H1NA	0.8537	C20B—C19B	1.390 (11)
N3A—C20A	1.130 (7)	N3X—C20X	1.138 (17)
C1A—C2A	1.493 (7)	C20X—C19B	1.372 (14)
C1A—C17A	1.549 (7)	C1B—C2B	1.503 (7)
C1A—H1AA	0.9900	C1B—C17B	1.542 (7)
C1A—H1AB	0.9900	C1B—H1BA	0.9900
C2A—C3A	1.519 (7)	C1B—H1BB	0.9900
C3A—C4A	1.538 (7)	C2B—C3B	1.517 (7)
C3A—C8A	1.567 (6)	C3B—C4B	1.537 (7)
C3A—H3AA	1.0000	C3B—C8B	1.537 (8)
C4A—C5A	1.516 (7)	C3B—H3BA	1.0000
C4A—H4AA	0.9900	C4B—C5B	1.529 (7)
C4A—H4AB	0.9900	C4B—H4BA	0.9900
C5A—C6A	1.520 (7)	C4B—H4BB	0.9900
C5A—H5AA	1.0000	C5B—C6B	1.507 (10)
C6A—C7A	1.541 (8)	C5B—H5BA	1.0000
C6A—H6AA	0.9900	C6B—C7B	1.534 (8)
C6A—H6AB	0.9900	C6B—H6BA	0.9900
C7A—C8A	1.539 (7)	C6B—H6BB	0.9900
C7A—H7AA	0.9900	C7B—C8B	1.531 (7)
C7A—H7AB	0.9900	C7B—H7BA	0.9900
C8A—C21A	1.543 (8)	C7B—H7BB	0.9900
C8A—C9A	1.545 (8)	C8B—C21B	1.544 (8)
C9A—C17A	1.515 (7)	C8B—C9B	1.561 (7)
C9A—C10A	1.536 (7)	C9B—C17B	1.540 (7)
C9A—H9AA	1.0000	C9B—C10B	1.546 (7)
C10A—C11A	1.547 (9)	C9B—H9BA	1.0000
C10A—H10A	0.9900	C10B—C11B	1.544 (7)
C10A—H10B	0.9900	C10B—H10C	0.9900
C11A—C12A	1.532 (8)	C10B—H10D	0.9900
C11A—H11A	0.9900	C11B—C12B	1.526 (7)
C11A—H11B	0.9900	C11B—H11C	0.9900
C12A—C16A	1.541 (6)	C11B—H11D	0.9900
C12A—C13A	1.543 (8)	C12B—C16B	1.542 (6)
C12A—C22A	1.544 (7)	C12B—C22B	1.547 (7)
C13A—C24A	1.516 (7)	C12B—C13B	1.555 (7)
C13A—C14A	1.568 (9)	C13B—C24B	1.535 (7)
C13A—H13A	1.0000	C13B—C14B	1.557 (7)
C14A—C15A	1.566 (7)	C13B—H13B	1.0000
C14A—H14A	0.9900	C14B—C15B	1.552 (7)
C14A—H14B	0.9900	C14B—H14C	0.9900
C15A—C16A	1.504 (9)	C14B—H14D	0.9900
C15A—H15A	0.9900	C15B—C16B	1.527 (7)
C15A—H15B	0.9900	C15B—H15C	0.9900
C16A—C17A	1.531 (8)	C15B—H15D	0.9900
C16A—H16A	1.0000	C16B—C17B	1.516 (7)
C17A—H17A	1.0000	C16B—H16B	1.0000
C18A—C19A	1.516 (7)	C17B—H17B	1.0000
C19A—C20A	1.463 (7)	C18B—C19B	1.517 (8)
C19A—H19A	0.9900	C19B—H19C	0.9900
C19A—H19B	0.9900	C19B—H19D	0.9900
C21A—H21A	0.9800	C19B—H19E	0.9900
C21A—H21B	0.9800	C19B—H19F	0.9900
C21A—H21C	0.9800	C21B—H21D	0.9800
C22A—H22A	0.9800	C21B—H21E	0.9800
C22A—H22B	0.9800	C21B—H21F	0.9800
C22A—H22C	0.9800	C22B—H22D	0.9800
C23A—C24A	1.537 (8)	C22B—H22E	0.9800
C23A—H23A	0.9800	C22B—H22F	0.9800
C23A—H23B	0.9800	C23B—C24B	1.528 (8)
C23A—H23C	0.9800	C23B—H23D	0.9800
C24A—C25A	1.536 (9)	C23B—H23E	0.9800
C24A—H24A	1.0000	C23B—H23F	0.9800
C25A—C26A	1.537 (8)	C24B—C25B	1.538 (7)
C25A—H25A	0.9900	C24B—H24B	1.0000
C25A—H25B	0.9900	C25B—C26B	1.528 (8)
C26A—C27A	1.528 (9)	C25B—H25C	0.9900
C26A—H26A	0.9900	C25B—H25D	0.9900
C26A—H26B	0.9900	C26B—C27B	1.507 (7)
C27A—C28A	1.517 (9)	C26B—H26C	0.9900
C27A—H27A	0.9900	C26B—H26D	0.9900
C27A—H27B	0.9900	C27B—C28B	1.544 (7)
C28A—C29A	1.501 (9)	C27B—H27C	0.9900
C28A—C30A	1.529 (9)	C27B—H27D	0.9900
C28A—H28A	1.0000	C28B—C30B	1.514 (8)
C29A—H29A	0.9800	C28B—C29B	1.514 (8)
C29A—H29B	0.9800	C28B—H28B	1.0000
C29A—H29C	0.9800	C29B—H29D	0.9800
C30A—H30A	0.9800	C29B—H29E	0.9800
C30A—H30B	0.9800	C29B—H29F	0.9800
C30A—H30C	0.9800	C30B—H30D	0.9800
Cl1B—C5B	1.818 (6)	C30B—H30E	0.9800
O1B—C18B	1.227 (6)	C30B—H30F	0.9800
			
C2A—N1A—N2A	119.0 (4)	N3B—C20B—C19B	172.9 (10)
C18A—N2A—N1A	119.4 (4)	N3X—C20X—C19B	171.0 (13)
C18A—N2A—H1NA	115.6	C2B—C1B—C17B	112.4 (4)
N1A—N2A—H1NA	124.7	C2B—C1B—H1BA	109.1
C2A—C1A—C17A	110.1 (4)	C17B—C1B—H1BA	109.1
C2A—C1A—H1AA	109.6	C2B—C1B—H1BB	109.1
C17A—C1A—H1AA	109.6	C17B—C1B—H1BB	109.1
C2A—C1A—H1AB	109.6	H1BA—C1B—H1BB	107.9
C17A—C1A—H1AB	109.6	N1B—C2B—C1B	128.7 (4)
H1AA—C1A—H1AB	108.2	N1B—C2B—C3B	116.2 (4)
N1A—C2A—C1A	129.7 (5)	C1B—C2B—C3B	115.1 (4)
N1A—C2A—C3A	116.9 (5)	C2B—C3B—C4B	112.2 (4)
C1A—C2A—C3A	113.2 (4)	C2B—C3B—C8B	112.7 (5)
C2A—C3A—C4A	113.7 (4)	C4B—C3B—C8B	114.0 (5)
C2A—C3A—C8A	108.3 (4)	C2B—C3B—H3BA	105.7
C4A—C3A—C8A	112.6 (4)	C4B—C3B—H3BA	105.7
C2A—C3A—H3AA	107.3	C8B—C3B—H3BA	105.7
C4A—C3A—H3AA	107.3	C5B—C4B—C3B	108.2 (5)
C8A—C3A—H3AA	107.3	C5B—C4B—H4BA	110.1
C5A—C4A—C3A	110.0 (4)	C3B—C4B—H4BA	110.1
C5A—C4A—H4AA	109.7	C5B—C4B—H4BB	110.1
C3A—C4A—H4AA	109.7	C3B—C4B—H4BB	110.1
C5A—C4A—H4AB	109.7	H4BA—C4B—H4BB	108.4
C3A—C4A—H4AB	109.7	C6B—C5B—C4B	113.1 (6)
H4AA—C4A—H4AB	108.2	C6B—C5B—Cl1B	109.0 (5)
C4A—C5A—C6A	110.3 (4)	C4B—C5B—Cl1B	107.9 (4)
C4A—C5A—Cl1A	109.9 (4)	C6B—C5B—H5BA	108.9
C6A—C5A—Cl1A	109.7 (4)	C4B—C5B—H5BA	108.9
C4A—C5A—H5AA	108.9	Cl1B—C5B—H5BA	108.9
C6A—C5A—H5AA	108.9	C5B—C6B—C7B	110.6 (5)
Cl1A—C5A—H5AA	108.9	C5B—C6B—H6BA	109.5
C5A—C6A—C7A	109.8 (5)	C7B—C6B—H6BA	109.5
C5A—C6A—H6AA	109.7	C5B—C6B—H6BB	109.5
C7A—C6A—H6AA	109.7	C7B—C6B—H6BB	109.5
C5A—C6A—H6AB	109.7	H6BA—C6B—H6BB	108.1
C7A—C6A—H6AB	109.7	C8B—C7B—C6B	113.7 (4)
H6AA—C6A—H6AB	108.2	C8B—C7B—H7BA	108.8
C8A—C7A—C6A	114.0 (4)	C6B—C7B—H7BA	108.8
C8A—C7A—H7AA	108.8	C8B—C7B—H7BB	108.8
C6A—C7A—H7AA	108.8	C6B—C7B—H7BB	108.8
C8A—C7A—H7AB	108.8	H7BA—C7B—H7BB	107.7
C6A—C7A—H7AB	108.8	C7B—C8B—C3B	108.1 (5)
H7AA—C7A—H7AB	107.7	C7B—C8B—C21B	108.6 (5)
C7A—C8A—C21A	109.5 (5)	C3B—C8B—C21B	110.7 (5)
C7A—C8A—C9A	111.2 (4)	C7B—C8B—C9B	110.7 (4)
C21A—C8A—C9A	111.4 (4)	C3B—C8B—C9B	108.4 (4)
C7A—C8A—C3A	106.2 (4)	C21B—C8B—C9B	110.4 (5)
C21A—C8A—C3A	110.8 (4)	C17B—C9B—C10B	111.0 (4)
C9A—C8A—C3A	107.6 (5)	C17B—C9B—C8B	111.7 (4)
C17A—C9A—C10A	109.6 (4)	C10B—C9B—C8B	114.8 (4)
C17A—C9A—C8A	113.8 (4)	C17B—C9B—H9BA	106.2
C10A—C9A—C8A	113.5 (5)	C10B—C9B—H9BA	106.2
C17A—C9A—H9AA	106.5	C8B—C9B—H9BA	106.2
C10A—C9A—H9AA	106.5	C11B—C10B—C9B	113.3 (4)
C8A—C9A—H9AA	106.5	C11B—C10B—H10C	108.9
C9A—C10A—C11A	111.5 (6)	C9B—C10B—H10C	108.9
C9A—C10A—H10A	109.3	C11B—C10B—H10D	108.9
C11A—C10A—H10A	109.3	C9B—C10B—H10D	108.9
C9A—C10A—H10B	109.3	H10C—C10B—H10D	107.7
C11A—C10A—H10B	109.3	C12B—C11B—C10B	112.3 (4)
H10A—C10A—H10B	108.0	C12B—C11B—H11C	109.1
C12A—C11A—C10A	113.7 (5)	C10B—C11B—H11C	109.1
C12A—C11A—H11A	108.8	C12B—C11B—H11D	109.1
C10A—C11A—H11A	108.8	C10B—C11B—H11D	109.1
C12A—C11A—H11B	108.8	H11C—C11B—H11D	107.9
C10A—C11A—H11B	108.8	C11B—C12B—C16B	106.8 (4)
H11A—C11A—H11B	107.7	C11B—C12B—C22B	110.3 (4)
C11A—C12A—C16A	106.0 (4)	C16B—C12B—C22B	111.8 (4)
C11A—C12A—C13A	116.3 (5)	C11B—C12B—C13B	117.9 (4)
C16A—C12A—C13A	100.3 (4)	C16B—C12B—C13B	99.8 (4)
C11A—C12A—C22A	110.1 (5)	C22B—C12B—C13B	109.8 (4)
C16A—C12A—C22A	112.4 (4)	C24B—C13B—C12B	119.7 (4)
C13A—C12A—C22A	111.4 (4)	C24B—C13B—C14B	111.5 (4)
C24A—C13A—C12A	121.2 (5)	C12B—C13B—C14B	103.5 (4)
C24A—C13A—C14A	111.8 (5)	C24B—C13B—H13B	107.2
C12A—C13A—C14A	102.7 (4)	C12B—C13B—H13B	107.2
C24A—C13A—H13A	106.8	C14B—C13B—H13B	107.2
C12A—C13A—H13A	106.8	C15B—C14B—C13B	106.4 (4)
C14A—C13A—H13A	106.8	C15B—C14B—H14C	110.5
C15A—C14A—C13A	106.2 (5)	C13B—C14B—H14C	110.5
C15A—C14A—H14A	110.5	C15B—C14B—H14D	110.5
C13A—C14A—H14A	110.5	C13B—C14B—H14D	110.5
C15A—C14A—H14B	110.5	H14C—C14B—H14D	108.6
C13A—C14A—H14B	110.5	C16B—C15B—C14B	104.2 (4)
H14A—C14A—H14B	108.7	C16B—C15B—H15C	110.9
C16A—C15A—C14A	103.4 (5)	C14B—C15B—H15C	110.9
C16A—C15A—H15A	111.1	C16B—C15B—H15D	110.9
C14A—C15A—H15A	111.1	C14B—C15B—H15D	110.9
C16A—C15A—H15B	111.1	H15C—C15B—H15D	108.9
C14A—C15A—H15B	111.1	C17B—C16B—C15B	118.2 (4)
H15A—C15A—H15B	109.0	C17B—C16B—C12B	114.6 (4)
C15A—C16A—C17A	119.7 (4)	C15B—C16B—C12B	104.8 (4)
C15A—C16A—C12A	104.1 (4)	C17B—C16B—H16B	106.1
C17A—C16A—C12A	115.0 (5)	C15B—C16B—H16B	106.1
C15A—C16A—H16A	105.7	C12B—C16B—H16B	106.1
C17A—C16A—H16A	105.7	C16B—C17B—C9B	109.7 (4)
C12A—C16A—H16A	105.7	C16B—C17B—C1B	110.4 (4)
C9A—C17A—C16A	108.2 (4)	C9B—C17B—C1B	111.5 (4)
C9A—C17A—C1A	112.1 (4)	C16B—C17B—H17B	108.4
C16A—C17A—C1A	109.8 (4)	C9B—C17B—H17B	108.4
C9A—C17A—H17A	108.9	C1B—C17B—H17B	108.4
C16A—C17A—H17A	108.9	O1B—C18B—N2B	122.2 (5)
C1A—C17A—H17A	108.9	O1B—C18B—C19B	121.3 (5)
O1A—C18A—N2A	123.3 (5)	N2B—C18B—C19B	116.5 (5)
O1A—C18A—C19A	122.6 (4)	C20X—C19B—C18B	112.6 (7)
N2A—C18A—C19A	114.0 (4)	C20B—C19B—C18B	120.6 (6)
C20A—C19A—C18A	112.4 (4)	C20B—C19B—H19C	107.2
C20A—C19A—H19A	109.1	C18B—C19B—H19C	107.2
C18A—C19A—H19A	109.1	C20B—C19B—H19D	107.2
C20A—C19A—H19B	109.1	C18B—C19B—H19D	107.2
C18A—C19A—H19B	109.1	H19C—C19B—H19D	106.8
H19A—C19A—H19B	107.8	C20X—C19B—H19E	109.1
N3A—C20A—C19A	175.5 (6)	C18B—C19B—H19E	109.1
C8A—C21A—H21A	109.5	C20X—C19B—H19F	109.1
C8A—C21A—H21B	109.5	C18B—C19B—H19F	109.1
H21A—C21A—H21B	109.5	H19E—C19B—H19F	107.8
C8A—C21A—H21C	109.5	C8B—C21B—H21D	109.5
H21A—C21A—H21C	109.5	C8B—C21B—H21E	109.5
H21B—C21A—H21C	109.5	H21D—C21B—H21E	109.5
C12A—C22A—H22A	109.5	C8B—C21B—H21F	109.5
C12A—C22A—H22B	109.5	H21D—C21B—H21F	109.5
H22A—C22A—H22B	109.5	H21E—C21B—H21F	109.5
C12A—C22A—H22C	109.5	C12B—C22B—H22D	109.5
H22A—C22A—H22C	109.5	C12B—C22B—H22E	109.5
H22B—C22A—H22C	109.5	H22D—C22B—H22E	109.5
C24A—C23A—H23A	109.5	C12B—C22B—H22F	109.5
C24A—C23A—H23B	109.5	H22D—C22B—H22F	109.5
H23A—C23A—H23B	109.5	H22E—C22B—H22F	109.5
C24A—C23A—H23C	109.5	C24B—C23B—H23D	109.5
H23A—C23A—H23C	109.5	C24B—C23B—H23E	109.5
H23B—C23A—H23C	109.5	H23D—C23B—H23E	109.5
C13A—C24A—C25A	109.8 (5)	C24B—C23B—H23F	109.5
C13A—C24A—C23A	112.6 (5)	H23D—C23B—H23F	109.5
C25A—C24A—C23A	110.1 (5)	H23E—C23B—H23F	109.5
C13A—C24A—H24A	108.1	C23B—C24B—C13B	113.1 (5)
C25A—C24A—H24A	108.1	C23B—C24B—C25B	109.4 (4)
C23A—C24A—H24A	108.1	C13B—C24B—C25B	110.5 (4)
C24A—C25A—C26A	116.4 (5)	C23B—C24B—H24B	107.9
C24A—C25A—H25A	108.2	C13B—C24B—H24B	107.9
C26A—C25A—H25A	108.2	C25B—C24B—H24B	107.9
C24A—C25A—H25B	108.2	C26B—C25B—C24B	114.6 (4)
C26A—C25A—H25B	108.2	C26B—C25B—H25C	108.6
H25A—C25A—H25B	107.3	C24B—C25B—H25C	108.6
C27A—C26A—C25A	112.1 (5)	C26B—C25B—H25D	108.6
C27A—C26A—H26A	109.2	C24B—C25B—H25D	108.6
C25A—C26A—H26A	109.2	H25C—C25B—H25D	107.6
C27A—C26A—H26B	109.2	C27B—C26B—C25B	112.3 (4)
C25A—C26A—H26B	109.2	C27B—C26B—H26C	109.1
H26A—C26A—H26B	107.9	C25B—C26B—H26C	109.1
C28A—C27A—C26A	115.5 (5)	C27B—C26B—H26D	109.1
C28A—C27A—H27A	108.4	C25B—C26B—H26D	109.1
C26A—C27A—H27A	108.4	H26C—C26B—H26D	107.9
C28A—C27A—H27B	108.4	C26B—C27B—C28B	114.9 (5)
C26A—C27A—H27B	108.4	C26B—C27B—H27C	108.5
H27A—C27A—H27B	107.5	C28B—C27B—H27C	108.5
C29A—C28A—C27A	110.0 (5)	C26B—C27B—H27D	108.5
C29A—C28A—C30A	110.4 (5)	C28B—C27B—H27D	108.5
C27A—C28A—C30A	111.7 (6)	H27C—C27B—H27D	107.5
C29A—C28A—H28A	108.2	C30B—C28B—C29B	110.3 (5)
C27A—C28A—H28A	108.2	C30B—C28B—C27B	111.0 (5)
C30A—C28A—H28A	108.2	C29B—C28B—C27B	111.9 (4)
C28A—C29A—H29A	109.5	C30B—C28B—H28B	107.8
C28A—C29A—H29B	109.5	C29B—C28B—H28B	107.8
H29A—C29A—H29B	109.5	C27B—C28B—H28B	107.8
C28A—C29A—H29C	109.5	C28B—C29B—H29D	109.5
H29A—C29A—H29C	109.5	C28B—C29B—H29E	109.5
H29B—C29A—H29C	109.5	H29D—C29B—H29E	109.5
C28A—C30A—H30A	109.5	C28B—C29B—H29F	109.5
C28A—C30A—H30B	109.5	H29D—C29B—H29F	109.5
H30A—C30A—H30B	109.5	H29E—C29B—H29F	109.5
C28A—C30A—H30C	109.5	C28B—C30B—H30D	109.5
H30A—C30A—H30C	109.5	C28B—C30B—H30E	109.5
H30B—C30A—H30C	109.5	H30D—C30B—H30E	109.5
C2B—N1B—N2B	118.5 (4)	C28B—C30B—H30F	109.5
C18B—N2B—N1B	118.3 (4)	H30D—C30B—H30F	109.5
C18B—N2B—H1NB	111.0	H30E—C30B—H30F	109.5
N1B—N2B—H1NB	129.6		
			
C2A—N1A—N2A—C18A	−178.8 (5)	N2B—N1B—C2B—C1B	−1.2 (9)
N2A—N1A—C2A—C1A	0.2 (8)	N2B—N1B—C2B—C3B	175.3 (5)
N2A—N1A—C2A—C3A	−175.8 (4)	C17B—C1B—C2B—N1B	−135.2 (6)
C17A—C1A—C2A—N1A	−118.9 (6)	C17B—C1B—C2B—C3B	48.2 (7)
C17A—C1A—C2A—C3A	57.3 (5)	N1B—C2B—C3B—C4B	0.6 (8)
N1A—C2A—C3A—C4A	−11.8 (6)	C1B—C2B—C3B—C4B	177.7 (5)
C1A—C2A—C3A—C4A	171.5 (4)	N1B—C2B—C3B—C8B	131.0 (5)
N1A—C2A—C3A—C8A	114.1 (5)	C1B—C2B—C3B—C8B	−51.9 (7)
C1A—C2A—C3A—C8A	−62.5 (5)	C2B—C3B—C4B—C5B	−173.3 (6)
C2A—C3A—C4A—C5A	−177.1 (4)	C8B—C3B—C4B—C5B	57.0 (7)
C8A—C3A—C4A—C5A	59.2 (5)	C3B—C4B—C5B—C6B	−56.1 (7)
C3A—C4A—C5A—C6A	−58.9 (6)	C3B—C4B—C5B—Cl1B	−176.7 (5)
C3A—C4A—C5A—Cl1A	179.9 (3)	C4B—C5B—C6B—C7B	55.3 (7)
C4A—C5A—C6A—C7A	57.8 (6)	Cl1B—C5B—C6B—C7B	175.3 (4)
Cl1A—C5A—C6A—C7A	179.0 (4)	C5B—C6B—C7B—C8B	−54.3 (7)
C5A—C6A—C7A—C8A	−58.2 (6)	C6B—C7B—C8B—C3B	53.3 (6)
C6A—C7A—C8A—C21A	−64.5 (6)	C6B—C7B—C8B—C21B	−66.8 (7)
C6A—C7A—C8A—C9A	172.0 (5)	C6B—C7B—C8B—C9B	171.8 (5)
C6A—C7A—C8A—C3A	55.3 (6)	C2B—C3B—C8B—C7B	174.8 (4)
C2A—C3A—C8A—C7A	177.9 (4)	C4B—C3B—C8B—C7B	−55.7 (6)
C4A—C3A—C8A—C7A	−55.5 (6)	C2B—C3B—C8B—C21B	−66.3 (6)
C2A—C3A—C8A—C21A	−63.3 (6)	C4B—C3B—C8B—C21B	63.1 (6)
C4A—C3A—C8A—C21A	63.4 (5)	C2B—C3B—C8B—C9B	54.9 (6)
C2A—C3A—C8A—C9A	58.7 (5)	C4B—C3B—C8B—C9B	−175.7 (4)
C4A—C3A—C8A—C9A	−174.6 (4)	C7B—C8B—C9B—C17B	−176.1 (5)
C7A—C8A—C9A—C17A	−171.9 (5)	C3B—C8B—C9B—C17B	−57.8 (6)
C21A—C8A—C9A—C17A	65.7 (6)	C21B—C8B—C9B—C17B	63.6 (6)
C3A—C8A—C9A—C17A	−56.0 (6)	C7B—C8B—C9B—C10B	56.3 (6)
C7A—C8A—C9A—C10A	61.9 (6)	C3B—C8B—C9B—C10B	174.6 (4)
C21A—C8A—C9A—C10A	−60.5 (6)	C21B—C8B—C9B—C10B	−63.9 (6)
C3A—C8A—C9A—C10A	177.8 (4)	C17B—C9B—C10B—C11B	50.9 (6)
C17A—C9A—C10A—C11A	56.8 (7)	C8B—C9B—C10B—C11B	178.8 (4)
C8A—C9A—C10A—C11A	−174.8 (5)	C9B—C10B—C11B—C12B	−54.1 (6)
C9A—C10A—C11A—C12A	−55.5 (7)	C10B—C11B—C12B—C16B	55.3 (5)
C10A—C11A—C12A—C16A	52.5 (7)	C10B—C11B—C12B—C22B	−66.3 (6)
C10A—C11A—C12A—C13A	162.9 (5)	C10B—C11B—C12B—C13B	166.5 (4)
C10A—C11A—C12A—C22A	−69.2 (6)	C11B—C12B—C13B—C24B	78.8 (6)
C11A—C12A—C13A—C24A	79.9 (7)	C16B—C12B—C13B—C24B	−166.2 (5)
C16A—C12A—C13A—C24A	−166.4 (5)	C22B—C12B—C13B—C24B	−48.7 (6)
C22A—C12A—C13A—C24A	−47.3 (7)	C11B—C12B—C13B—C14B	−156.4 (4)
C11A—C12A—C13A—C14A	−154.5 (5)	C16B—C12B—C13B—C14B	−41.3 (5)
C16A—C12A—C13A—C14A	−40.9 (5)	C22B—C12B—C13B—C14B	76.2 (5)
C22A—C12A—C13A—C14A	78.2 (5)	C24B—C13B—C14B—C15B	152.8 (4)
C24A—C13A—C14A—C15A	151.3 (5)	C12B—C13B—C14B—C15B	22.8 (5)
C12A—C13A—C14A—C15A	19.8 (6)	C13B—C14B—C15B—C16B	5.2 (6)
C13A—C14A—C15A—C16A	9.7 (6)	C14B—C15B—C16B—C17B	−161.0 (4)
C14A—C15A—C16A—C17A	−166.1 (5)	C14B—C15B—C16B—C12B	−31.9 (5)
C14A—C15A—C16A—C12A	−36.0 (6)	C11B—C12B—C16B—C17B	−59.9 (5)
C11A—C12A—C16A—C15A	170.2 (5)	C22B—C12B—C16B—C17B	60.9 (6)
C13A—C12A—C16A—C15A	48.8 (5)	C13B—C12B—C16B—C17B	176.9 (4)
C22A—C12A—C16A—C15A	−69.5 (6)	C11B—C12B—C16B—C15B	169.0 (4)
C11A—C12A—C16A—C17A	−57.0 (6)	C22B—C12B—C16B—C15B	−70.3 (5)
C13A—C12A—C16A—C17A	−178.3 (4)	C13B—C12B—C16B—C15B	45.8 (5)
C22A—C12A—C16A—C17A	63.3 (6)	C15B—C16B—C17B—C9B	−176.7 (4)
C10A—C9A—C17A—C16A	−58.3 (6)	C12B—C16B—C17B—C9B	58.9 (6)
C8A—C9A—C17A—C16A	173.4 (4)	C15B—C16B—C17B—C1B	−53.5 (6)
C10A—C9A—C17A—C1A	−179.5 (5)	C12B—C16B—C17B—C1B	−177.8 (4)
C8A—C9A—C17A—C1A	52.2 (6)	C10B—C9B—C17B—C16B	−51.9 (5)
C15A—C16A—C17A—C9A	−173.2 (5)	C8B—C9B—C17B—C16B	178.5 (4)
C12A—C16A—C17A—C9A	61.7 (5)	C10B—C9B—C17B—C1B	−174.5 (4)
C15A—C16A—C17A—C1A	−50.6 (6)	C8B—C9B—C17B—C1B	55.9 (6)
C12A—C16A—C17A—C1A	−175.7 (4)	C2B—C1B—C17B—C16B	−172.0 (4)
C2A—C1A—C17A—C9A	−50.5 (6)	C2B—C1B—C17B—C9B	−49.8 (6)
C2A—C1A—C17A—C16A	−170.8 (4)	N1B—N2B—C18B—O1B	179.9 (5)
N1A—N2A—C18A—O1A	−178.7 (5)	N1B—N2B—C18B—C19B	−1.6 (8)
N1A—N2A—C18A—C19A	4.2 (7)	O1B—C18B—C19B—C20X	31.8 (10)
O1A—C18A—C19A—C20A	−4.5 (8)	N2B—C18B—C19B—C20X	−146.7 (7)
N2A—C18A—C19A—C20A	172.6 (5)	O1B—C18B—C19B—C20B	−129.0 (7)
C12A—C13A—C24A—C25A	179.5 (5)	N2B—C18B—C19B—C20B	52.5 (9)
C14A—C13A—C24A—C25A	58.2 (6)	C12B—C13B—C24B—C23B	−52.0 (7)
C12A—C13A—C24A—C23A	−57.5 (7)	C14B—C13B—C24B—C23B	−173.0 (5)
C14A—C13A—C24A—C23A	−178.8 (5)	C12B—C13B—C24B—C25B	−175.0 (5)
C13A—C24A—C25A—C26A	−173.5 (5)	C14B—C13B—C24B—C25B	64.0 (6)
C23A—C24A—C25A—C26A	61.9 (7)	C23B—C24B—C25B—C26B	72.1 (6)
C24A—C25A—C26A—C27A	173.2 (6)	C13B—C24B—C25B—C26B	−162.7 (5)
C25A—C26A—C27A—C28A	−178.8 (6)	C24B—C25B—C26B—C27B	−162.9 (5)
C26A—C27A—C28A—C29A	173.3 (6)	C25B—C26B—C27B—C28B	178.1 (4)
C26A—C27A—C28A—C30A	−63.6 (8)	C26B—C27B—C28B—C30B	−168.9 (5)
C2B—N1B—N2B—C18B	177.9 (5)	C26B—C27B—C28B—C29B	67.3 (6)

### Hydrogen-bond geometry (Å, º)

**Table:**

*D*—H···*A*	*D*—H	H···*A*	*D*···*A*	*D*—H···*A*
C4*B*—H4*BA*···N3*B*	0.99	2.58	3.469 (10)	149
N2*A*—H1*NA*···O1*B*^i^	0.85	2.03	2.870 (6)	168
N2*B*—H1*NB*···O1*A*^ii^	0.85	2.05	2.894 (6)	171
C1*A*—H1*AB*···O1*B*^i^	0.99	2.41	3.362 (7)	162
C1*B*—H1*BB*···O1*A*^ii^	0.99	2.44	3.352 (6)	153
C4*A*—H4*AB*···N3*B*^iii^	0.99	2.49	3.476 (9)	173
C19*A*—H19*A*···N3*B*^iii^	0.99	2.57	3.513 (11)	160

Symmetry codes: (i) −*x*+1, *y*+1/2, −*z*+1/2; (ii) −*x*+1, *y*−1/2, −*z*+1/2; (iii) −*x*, *y*+1/2, −*z*+1/2.
